# Radial Basis Function Artificial Neural Network for the Investigation of Thyroid Cytological Lesions

**DOI:** 10.1155/2020/5464787

**Published:** 2020-11-24

**Authors:** Christos Fragopoulos, Abraham Pouliakis, Christos Meristoudis, Emmanouil Mastorakis, Niki Margari, Nicolaos Chroniaris, Nektarios Koufopoulos, Alexander G. Delides, Nicolaos Machairas, Vasileia Ntomi, Konstantinos Nastos, Ioannis G. Panayiotides, Emmanouil Pikoulis, Evangelos P. Misiakos

**Affiliations:** ^1^Department of Cytopathology, Kavala General Hospital, Region of Eastern Macedonia and Thrace, Greece; ^2^2nd Department of Pathology, National and Kapodistrian University of Athens, “Attikon” University Hospital, Athens, Greece; ^3^Klinik Diagnostik, Aalborg University Hospital, Aalborg, Denmark; ^4^Department of Cytopathology, Venizeleion General Hospital, Heraklion, Crete, Greece; ^5^Department of Pathology, Venizeleion General Hospital, Heraklion, Crete, Greece; ^6^2nd Department of Otolaryngology—Head and Neck Surgery, National and Kapodistrian University of Athens, “Attikon” University Hospital, Athens, Greece; ^7^3rd Department of Surgery, National and Kapodistrian University of Athens, “Attikon” University Hospital, Athens, Greece

## Abstract

**Objective:**

This study investigates the potential of an artificial intelligence (AI) methodology, the radial basis function (RBF) artificial neural network (ANN), in the evaluation of thyroid lesions. *Study Design*. The study was performed on 447 patients who had both cytological and histological evaluation in agreement. Cytological specimens were prepared using liquid-based cytology, and the histological result was based on subsequent surgical samples. Each specimen was digitized; on these images, nuclear morphology features were measured by the use of an image analysis system. The extracted measurements (41,324 nuclei) were separated into two sets: the training set that was used to create the RBF ANN and the test set that was used to evaluate the RBF performance. The system aimed to predict the histological status as benign or malignant.

**Results:**

The RBF ANN obtained in the training set has sensitivity 82.5%, specificity 94.6%, and overall accuracy 90.3%, while in the test set, these indices were 81.4%, 90.0%, and 86.9%, respectively. Algorithm was used to classify patients on the basis of the RBF ANN, the overall sensitivity was 95.0%, the specificity was 95.5%, and no statistically significant difference was observed.

**Conclusion:**

AI techniques and especially ANNs, only in the recent years, have been studied extensively. The proposed approach is promising to avoid misdiagnoses and assists the everyday practice of the cytopathology. The major drawback in this approach is the automation of a procedure to accurately detect and measure cell nuclei from the digitized images.

## 1. Introduction

Cytopathology, a medical discipline born in the 20^th^ century, was founded by George Papanicolaou in 1928 [1] and became very popular due to the worldwide known Papanicolaou test [2, 3] (test Pap). Although cervical cancer represents the vast majority of cytological diagnoses worldwide, it is not the only disease that cytopathology deals with. Even in the early days of cytopathology [4–6], it was used for the investigation of thyroid gland and body fluids. One of the main advantages of thyroid cytopathology is its noninvasive or minimally invasive nature. That is, the biological material is extracted from patients with nonpainful methods (for example, cells are extracted using a fine needle).

Cytopathology of the thyroid gland is a well-documented method, extensively used for preoperative diagnosis of the thyroid nodules [7, 8]. The Bethesda System for reporting thyroid cytopathology (TBS [7]) is a well-established system for the evaluation of thyroid lesions [9–11] with link to the risk for malignancy guiding clinicians towards patient management. TBS guides cytopathologists in the classification of thyroid lesions via established criteria.

The diagnostic accuracy of thyroid fine-needle aspiration (FNA) has been reported by many studies as having both high sensitivity (80%–90.8% [12, 13]) and specificity (60%–100% [14–16]). Despite, TBS is already widely used in various countries, and it has its own limitations [17–19], especially due to gray diagnostic zones. Thus, false-positive or false-negative results can be observed not only due to poor cellular smears but also misinterpretation of inadequate representation of the morphological characteristics of the smear, especially in the case of follicular neoplasms. Admitted, the experience of the individual cytopathologist, in both the particular organ and the general diagnostic experience, is an important factor in a proper diagnosis and is based mainly on extensive training.

During the last decade, information technology and artificial intelligence (AI) enabled the creation of computer-aided systems supporting diagnosis as well as decisions for therapy and patient management. Among the numerous machine learning methodologies aiming towards the solution of such medical tasks are artificial neural networks (ANNs) [20–32], more classical approaches such as discriminant analysis [20, 33, 34], classification, and regression trees [35–38], genetic algorithms [39], and in the last decade, deep learning [40–42]. Such techniques are not new in the diagnostic cytopathology field [43], since they have been already employed in diagnostic tasks for numerous organs such as the stomach [33, 44, 45], breast [46–49], urinary system [50–52], cervix [53–57], and thyroid [58–61] among others.

Given that a gray zone in the thyroid cytopathology classification system exists, in this study, we focus on the investigation of the potential of a rarely used ANN (namely, the radial basis function network—RBF [51]) into the classification of thyroid specimens based on cytomorphological characteristics.

## 2. Materials and Methods

The study was performed in FNA specimens that had a follow-up of a histological evaluation from thyroidectomy specimens (performed at the 2^nd^ Department of Pathology, National and Kapodistrian University of Athens, Medical School, “Attikon” University Hospital). The study was carried out on cytological slides collected from 2012 to 2016 and conformed to the principles of the Helsinki Declaration. In addition, it was approved by the Ethics Review Board of “Attikon” University Hospital, and the requirement of a signed consent form was waived, since it was based on archived material and had no invasive or treatment effects on the patients.

We analyzed liquid-based cytology (LBC) specimens from 447 patients; from each specimen, various cell types were isolated and characterized.  Table 1 shows the confusion matrix (histological diagnosis vs. cells measured in the cytological slides). All cases were selected in a serial manner from the database, and cases without histological confirmation were excluded from the study. In a total 288 cases, the lesion was benign (64.4%), and in the remaining 159 cases (35.6%), it was malignant. We followed the methods of an approach proposed by Margari et al. in 2019 [62] especially for the image analysis and the subsequent construction of the ANN system.

### 2.1. Liquid-Based Cytology (LBC)

Liquid-based cytology (LBC) was used due to the offered advantages over conventional cytopathology, i.e., reduction of air drying artifacts, blood and inflammation obscuring the cells reduction, distribution of the cells in a single layer on the slide in a smaller area than the conventional allowing rapid screening, well preserved nuclear details, and the possibility to obtain additional slides and/or biological material for molecular tests and immunocytochemical staining. Aspirates were immersed and rinsed into a vial containing CytoLyt® (fixative solution). Subsequently, a single slide was prepared, and the ThinPrep® technique was applied, as already described [63]. Cytology diagnoses were formulated according to the TBS system [7, 9–11].

### 2.2. Architecture of the AI System

**Figure 1:**
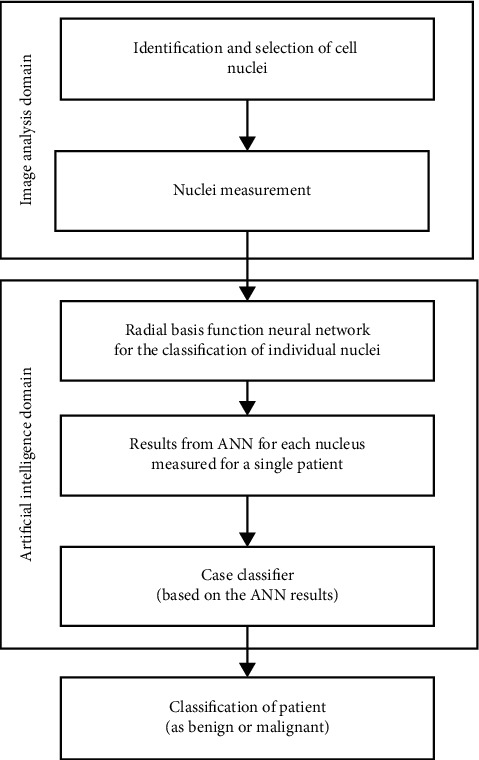
Flow chart of the system architecture.

A system with the architecture depicted in Figure 1 was created to classify each individual patient as benign or malignant by the use of measurements from the cytological slides. Two different technologies were used for this application: (a) image analysis which involves the selection of cell nuclei from the digitized images and subsequently their measurement; from this step is a series of measurements characteristic for each cell nucleus extracted, and (b) the second technological domain is relevant to artificial intelligence; in this part of the system, individual nucleus was classified as benign or malignant by the RBF ANN, which produces a list of classified nuclei for each cytological slide; this information is subsequently fed to a second subsystem (the case classifier), which can identify individual patients as belonging to the benign or malignant group. The case classifier can be based on a majority logic methodology either for the number of nuclei within each group or their percentage [58, 64, 65].

### 2.3. Image Analysis and Nucleus Morphometry

A manual selection process was applied in order to select representative nuclei from every cytological slide. This manual selection process ranges between 10 and 20 minutes per slide and consists of the definition of the nucleus borders via the mouse; note that for each patient, a single cytological slide is prepared. Subsequently, every nucleus was measured by the computer within milliseconds (note that this is a batch process that operates massively on identified nuclei). Detailed information for extracted nuclear morphometrical features are summarized in Table 2. The measurement algorithms have been already reported in the literature [58, 66–68], and descriptive characteristics have already been reported in our previous article [65].

For image analysis purposes, a computer equipped with a frame grabber and a digital camera (SONY DFW-X700, Sony Corporation, Tokyo, Japan) was used. A microscope (Leica Microsystems GmbH, Wetzlar, Germany) had the camera attached via a c-mount adaptor and was interfaced via the appropriate cables to the frame grabber (installed in the computer). The images were captured using a 40x objective and digitized by the frame grabber into 1024 × 768 pixels (8 bits for each color component (red green and blue, i.e., 24 bits of depth).

PathSight version 4.3 (Medical Solutions PLC, UK) was used to capture the images, and Image-Pro Plus VERSION 4.5 (Media Cybernetics, Inc. Bethesda, MD, USA) was used for the isolation of nuclei (segmentation) and subsequent measurement (morphometry). Moreover, Image-Pro Plus was used for background correction and to remove the noise caused by the lenses of the microscope, i.e., to alleviate noise caused from dust particles interfering in the light path and calibration for lighting, ensuring that all images were captured under similar conditions, and therefore, there was improved reproducibility of results and quality control.

**Figure 2:**
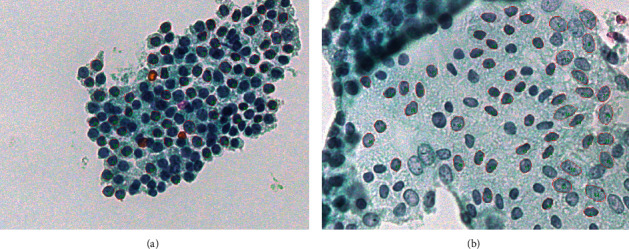
Typical images from the cytological material with highlighted selected nuclei. (a) Image of nodular hyperplasia (benign); (b) image of papillary carcinoma. Both images were captured using a 40x microscope objective lens.

The measurements obtained can be categorized into two types: (a) geometric and (b) densitometric [32, 64, 65, 67, 69–75]. In general, geometric and densitometric can be considered unrelated, as geometric features are based on the nucleus boundary and are relevant to nucleus shape characteristics. Carcinogenesis causes destruction effects of the nucleus—skeleton and cytoskeleton; therefore, nuclei are deformed, and this is reflected in geometric characteristics. The densitometric characteristics are extracted from pixel values and their spatial distribution within the nucleus boundary. In detail, the nucleus boundary is represented as a polygon, and the coordinates of the edge points of this polygon are used to extract geometric characteristics; for example, the nucleus area is calculated as the sum of the triangles that compose the nucleus surrounding polygon. More details on calculation algorithms can be found in the relevant bibliography for image analysis [69, 72]. A characteristic image is presented in Figure 2.

### 2.4. Measured Cytological Structures Training and Test Sets

The total number of selected cell nuclei (and colloid structures) was 41,324 (Table 3); they were picked either from cell groups or they were found isolated in the field of view.

About 50% (*N* = 224) of patients were randomly selected to form the training set; thus, the measured cytological structures from these cases were used to train the AI system. The remaining cases formed the test set and were used to evaluate the performance of the system on unknown data and therefore validate the results.

Since the dataset is not balanced between the classes (288 cases were benign and 159 cases were malignant, i.e., 64% and 36%, respectively), the random selection of 50% of the cases to form the training set, eventually, lead to a representative selection of the cases respecting this imbalanced distribution. Actually, the training set was composed of 145 benign and 79 malignant cases (65% and 35%) and the test set of 143 and 80 cases (i.e., 64% and 36%, respectively). Note that during the training stage of the ANN, it is important to respect the data distribution within different classes to avoid learning towards one direction.

Nowadays, the most popular percentages used to separate the data into training and test sets are 70%–30% or even 80%–20%, following the Pareto principle [76]. In this study, we preferred to use 50% of the data for training and the remaining for test for several reasons: (a) we have already applied successfully this approach in numerous other cytology-related classification problems in the past, (b) there were a lot of data available, especially in the nucleus classification domain; thus, we expected that the data variance would be possible to be “learned” by the 50% of the available data, (c) less data in the training set reduces the probability for overfitting (i.e., the ANN learns extremely well the training set but fails to perform well in the test set), and (d) we preferred to have almost equal samples in the training and test sets in order to compare the performance in these two sets and therefore assess the system robustness.

### 2.5. The Radial Basis Function Artificial Neural Network

Artificial neural networks are mathematical models mimicking the human brain structure. They are capable to learn and subsequently recall patterns [43, 77, 78]; thus, they are ideal to learn the nuclear patterns as these are represented through the measurements and subsequently assign the nuclei to individual categories (benign or malignant in this study).

A radial basis function network (RBF) uses radial basis functions as activation functions; they have strictly three layers: (a) an input layer that serves after weighting all the measurements in all nodes of the subsequent layer (the hidden layer), (b) the hidden layer that implements a series of nonlinear RBF activation functions responsible to create clusters of similar data, and (c) the linear output layer that is actually a linear combination of radial basis functions results from the inputs and the neuron parameters and creates the ANN output (i.e., classification result). Despite RBF, ANNs are here for more than 30 years (first presented in 1988 [79]) and have many uses, such as function approximation, time series prediction, classification, and control; among others, they have not been used extensively in medical applications. However, they do possess some advantages compared to classical architectures such as (a) the multilayer perceptrons have faster training, (b) it is possible to interpret what is the role of the nodes in the hidden layer, and (c) the number of nodes in the hidden layer (RBF nodes) is adjusted from the data.

### 2.6. Classification for the Patients

Cell nuclei classification by the RBF ANN cannot on its own assign patients as having benign or malignant thyroid disease. Thus, an additional subsystem was incorporated according to a technique already reported in the literature [58, 64, 65]. Specifically, two different approaches were used called subsequently the numeric and percentages classifiers; these assign a case as benign if a number or percentage of nuclei, respectively, classified as benign by the RBF ANN is above a certain threshold, otherwise as malignant. In order to find such threshold, we evaluated the specificity and sensitivity in a broad range of thresholds starting from 1 (or 1%) and increasing up to 100 (or 100%) with an increment step of 1 (or 0.1). For every value, the percentage of the cases that have been correctly classified was calculated as well as the sensitivity and specificity. As the most suitable threshold, the threshold that produced a balanced result between sensitivity and specificity (i.e., minimized their difference) was used. In order to avoid bias, only the nuclei classified in the training set were used to find these thresholds.

### 2.7. Tools and Techniques

The RBF ANN for nuclei classification and the algorithms for the determination of the optimum thresholds were constructed with in-house developed software for the MATLAB environment (The MathWorks, Inc. Natick, Massachusetts, U.S.A.). Moreover, MATLAB was used to calculate the performance indicators for the training and test sets and all data combined. Statistical measures used were specificity, sensitivity, positive and negative predictive value (PPV and NPV), false-positive and false-negative rates (FPR and NPR), overall accuracy (OA), and odds ratio. A list of the performance indices along with the mathematical formulas and a short description is already reported [80].

## 3. Results

### 3.1. Results of Cell Nuclei Classification

The performance of the RBF ANN was evaluated for the training set, the test set, and the complete data set (training and test sets combined). The results are presented in Table 4.

As expected, the performance (Table 5) was better in the training set. Comparison of proportions (*z*-test) revealed that a statistically significant difference was present in (a) sensitivity (difference: 1.14%, 95% CI: 0.40%–1.89%, *p* = 0.0027), (b) specificity (difference: 4.58%, 95% CI: 4.07%–5.10%, *p* < 0.0001), (c) positive-predictive value (difference: 7.74%, 95% CI: −7.06%–8.42%, *p* < 0.0001), (d) negative-predictive value (difference 0.87%, 95% CI: 0.29%–1.45%, *p* = 0.0031), (e) false-positive rate (difference: 4.58%, 95% CI: 4.07%–5.10%, *p* < 0.0001), (f) false-negative rate (difference: 1.14%, 95% CI: 0.40%–1.89%, *p* = 0.0027), and (g) overall accuracy (difference: 3.32%, 95% CI: −2.71%–3.94%, *p* < 0.0001). Note that due to the large number of nuclei, even small differences in the percentages can lead to statistically significant differences.

### 3.2. Results of Patient Classification

As mentioned, two approaches were used for the classification of patients: the numeric classifier and the percentages classifier, and the results of these two different methodologies are presented in Table 6, and the relevant performance indices are presented in Table 7. Specifically, the threshold that produced the more balanced results between sensitivity and specificity for the numeric classifier was 37 nuclei, i.e., if more than 37 nuclei were classified by the RBF ANN as benign, then the case (i.e., the patient) was classified as benign, otherwise as malignant. In a similar approach, the threshold for the percentages classifier was 51%, i.e., if more than 51% of the case nuclei were classified by the RBF ANN as benign, then the sample (the patient) was considered as benign, otherwise as malignant.

**Figure 3:**
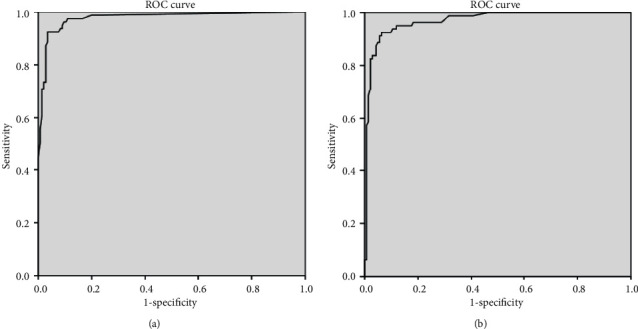
Receiver operating characteristic (ROC) curves for the numeric classifier: (a) the training set and (b) the test set.

Notably, the percentages classifier had better indices for the three most important metrics: sensitivity, specificity, and overall accuracy. And in general, these indicators were lower in the test set. In order to test the stability of the approach, we performed comparisons (*z*-test) for the sensitivity, specificity, and overall accuracy between the training and test sets; for the numeric classifiers, the difference, 95% CI, and *p* values were 1.16%, 95% CI: −4.25–6.63, and *p* = 0.7833; 0.77%, 95% CI: −4.0–5.57, and *p* = 0.8850, and 0.92%, 95% CI: −4.1–5.98, and *p* = 0.8417, respectively. Similarly, for the percentages classifier, there was no observed statistically significant difference. Thus, both approaches can be considered as stable. This is also reflected by the comparison of the areas under curve for the receiver operating characteristic (ROC) curves between the training and the test sets (Figure 3). Specifically, for the arithmetic classifier, the area under curve (AUC) for the training and test sets was 97.7% (95% CI: 95.7%–99.7%) and 96.9% (95% CI: 94.8%–99.1%), respectively, and no statistically significant difference was confirmed (*p* > 0.05). Similarly, for the percentages classifier, the AUC for the training and test sets was 98.1% (95% CI: 96.2%–100%) and 98.1% (95% CI: 96.6%–99.7%), respectively, and no statistical difference was possible to be confirmed (*p* > 0.05). Moreover, we compared the same three performance indices between the numeric and percentages classifier (considering the training, test, and all data combined); again, no statistically significant difference was proved (*p* > 0.05 for all comparisons). In summary, both case classifiers can be considered stable between the training and test sets and of similar performance.

## 4. Discussion

The history of artificial intelligence in thyroid cytopathology is really worth to investigate, so that the evolution of the various efforts can be highlighted. Thyroid disease and ANNs first appeared to the authors' knowledge, in 1993 [81], and a few years later, the first application in cytopathology appeared in 1996 [27]. Specifically, it used the backpropagation training algorithm to train a feedforward three-layer (one input, one hidden, and one output layers) ANN. This ANN was discriminating between benign from malignant thyroid nuclei, according to nuclear morphometry. The number of patients in this article was rather small (51). At the nuclei classification level, an overall accuracy of 90.6% was achieved, and classification of individual patients had an overall accuracy of 98%. Three years later, in 1999 [61], four variations of the LVQ classifier (namely, the versions LVQ1, LVQ2.1, LVQ3, and OLVQ1-optimized LVQ) were tested in 100 patients. A different approach was used, i.e., the mean value and standard deviation of nucleus morphometry features were employed for each patient, and in contrast to this approach, the ANN is applied on individual nuclei; thus, the patients were represented from the statistics measures of the cell nuclei. These LVQ variations enabled classification of 97.7% benign vs. malignant patients, but no important results finer classification in the histological subgroups was reported or obtained. In 2006 [31], Cochand-Priollet et al. reported on the application of four different classification methods after nuclear morphometry that was followed by statistical preselection in order to identify the significant features (notably only four image morphometry features were different and were considered important for subsequent classification). Four classifiers were compared: (1) a linear classifier, (2) a two-layer feedforward, (3) a combined two-layer feedforward ANNS generated by the Ada–Boost method, and (4) the *k* nearest neighbor classifier (a method with many similarities with LVQ). The results of the classifiers were between 83% and 94%, with the linear classifier having the worst performance (65%) in patient discrimination. The latter proved that ANNs can exploit their nonlinear nature to obtain better classification results than typical statistical approaches such as the linear models.

In the same arena, in 2007, Shapiro et al. [82] used 197 thyroid follicular tumors (adenomas and carcinomas); various types of ANNs and different designs were tested using nuclear morphometry (i.e., area, perimeter, and shape factor) and nuclear density or texture features (mean value and standard deviation of gray levels). In a similar approach, the ANNs were applied on the mean values of the nuclei measurements; thus, each patient was represented by a single vector of values. According to the results, the diagnostic accuracy in detecting follicular tumors was 97%, and the accuracy of ANNs in discriminating adenomas from carcinomas was by 87%. This research team reported that the application of ANNs raised the sensitivity of cytological diagnosis of follicular tumors to 90%, while at these times, the usual cytopathology approach had sensitivity around 60%.

The problem of indeterminate results of thyroid cytopathology was first reported in 2004 [83], whereas in a large group of patients (*N* = 453), a feedforward ANN was trained and tested using not only cytopathology results but additionally combined with clinical data. Patients were separated into high or low risk for malignancy and reported that only the cytological parameters contributed towards this classification. In this study, it was reported that there was no difference between the training and the test set results; thus, the method was not only important in the gray zone of cytopathology of the thyroid but also considered as robust (note that usually the robustness test is not reported in the various studies).

In 2006, two important articles presented [84, 85] with several novelties: (a) a two-layer ANN was employed with the layer having one input assigned to one training image, (b) the classification was based on image frequency bands (i.e., application of the Fourier transform in the two-dimensional domain); thus, the novelty was that no complex morphometry operations were required and most interestingly, the cell identification (an extremely difficult task), and (c) the proposed system discriminated the cases as follicular carcinomas, follicular adenomas, or unknown; thus, the ANN was capable to handle cases impossible to discriminate. Notably, this approach introduces a gray zone quite shorter than the cytological.

The first combinatorial approach was presented by Daskalakis et al., [86] in 2008, who applied a system composed of multiple classifiers in order to discriminate benign from malignant thyroid nodules. The team used an ensemble of classifiers and applied combinations of rules in the classifiers involved. Similarly, to the majority of the efforts, this study used nuclear morphological features. The classification results were in the range of 95.7%, while the best single classifier had an accuracy of 89.6%. Therefore, a new combinatorial methodology for thyroid cytopathology and ANNS was first introduced and had the potential for better accuracy from a single ANN. Similarly, in 2011 [58], the application of a combinatorial approach of two ANNs, one for nuclei classification and a cascaded second ANN for patient classification based on the LVQ and monolayer smears, was reported. The study had relatively a large number of patients (*N* = 335). These two combined ANNs had an overall accuracy of 94%. The study concluded that the diagnostic accuracy of thyroid FNA can be improved by the use of ANNs. More interesting results were for follicular neoplasms suspicious for malignancy and in Hürthle cell tumors.

Finally, there are approaches that are based on histological sections. For example, in 2014 [87], Ozolek et al. presented a method distinguishing follicular thyroid lesions using the optimal transport-based linear embedding to segment cell nuclei [88]. The results of the classification were almost perfect, and the classification was based on isolated nuclei using a supervised method [89].

Finally, in the recent years, ANNs have been again in the front line, and there are more efforts reported, for example, to distinguish follicular adenomas from follicular carcinomas [90] or papillary carcinomas [91]. Interestingly, whole slide imaging applications started to appear, and deep learning approaches have been introduced [92, 93].

Since there is no standardization on the reported results, for example, some reports mention sensitivity or specificity and others overall accuracy, it is extremely difficult to have comparative results of the various approaches. Moreover, the classification unit differs, for example, in some reports, the discrimination is on patients, while in others, on images and even at the cell nucleus level. Finally, the classification domain can be between benign or malignant lesions and in other reports between follicular adenomas vs. follicular carcinomas. For completeness reasons, Table 8 presents the various approaches and the applied classification technique, along with the classification units, domain, and performance.

This study has an important novelty; it represents the first approach that the RBF ANN, a rarely applied ANN in the field of medicine, is used to discriminate benign from malignant thyroid patients on the basis of image morphometry in monolayer cytological slides. The results indicated that the proposed system was robust as compared between the training and test sets, for both patient classification approaches, while the performance was in the range of the performance indicators reported by the other studies so far conducted.

The major advantages and novelty of this approach can be summarized as follows: (a) there is increased objectivity in the method since there are measurable features, (b) the slide preparation using the single layer approach and staining devices contributes towards this objectivity, (c) the diagnostic accuracy if we consider only the cytological examination seems increased; however, this is a subjective measure since it depends on the laboratory performing the cytological examinations, and (d) the decision mechanism is based on computers and is not dependent on human factors; however, note that specialized cytopathologists are required to define the nucleus borders required for the image measurements process.

More efforts seem to be needed towards the automation of the procedure, since only a small number of nuclei (about 100/patient) are used both in this study and in other studies. It seems that the application of whole slide imaging and a simultaneous detection of higher number of nuclei would be of interest. Moreover, automated cell nuclei identification and measurement seem to be of interest for further research. This approach highlighted a new combined methodology for thyroid cytopathology that has the potential to evolve to greatest accuracy and automation.

## Figures and Tables

**Table 1:** Cases used in the study grouped as benign or malignant according to the histological diagnosis (rows). In the columns, the cytological results are indicated.

Histology	Colloid	Follicular cells	Follicular neoplasm	Histiocytes	Lymphocytes	Oxyphilic cells	Anaplastic Ca	Follicular Ca	Medullary Ca	Papillary Ca	Total
Benign	26	236	4	6	7	9					288
Adenomatous nodule	2	22									24
Follicular adenoma	1		4								5
Goiter	20	159		3		4					186
Hashimoto thyroiditis		13			7	4					24
Nodular hyperplasia		3									3
Nodular hyperplasia-hyperplastic nodule		37									37
Oxyphilic adenoma	2			1		1					4
Thyroiditis-nonspecific	1	2		2							5

Malignant		3		5	2		3	10	20	116	159
Anaplastic Ca							3				3
Follicular Ca								10			10
Medullary Ca									20		20
Papillary Ca		3		5	2					116	126

Total	26	239	4	11	9	9	3	10	20	116	447

Ca, carcinoma.

**Table 2:** Image analysis measurements categorized into geometrical and densitometric.

Geometric characteristics	Densitometric characteristics
Nucleus area	Integrated optical density
Nucleus major axis	Mean value of nucleus red color
Nucleus minor axis	Mean value of nucleus green color
Aspect ratio	Mean value of nucleus blue color
Maximum calliper	Mean value of optical density
Minimum calliper	Maximum value of optical density
Average value of calliper	Minimum value of optical density
Maximum nucleus radius	Standard deviation of optical density
Minimum nucleus radius	Margination
Radius ratio	Heterogeneity
Nucleus perimeter	
Nucleus roundness	
Fractal dimension	

**Table 3:** Cell nuclei and colloid structures measured from the cytological slides in relation to the histological result.

Histology	Colloid	Follicular neoplasm	Histiocytes	Lymphocytes	Oxyphilic cells	Stromal cells	Follicular cells	Medullary Ca	Anaplastic Ca	Papillary Ca	Follicular Ca	Total
Benign	250	388	1134	217	2291	2	22308	3		3		26596
Adenomatous nodule	2		147	8			2057					2214
Follicular adenoma	4	388	3									395
Goiter	209		863		820	2	15370			3		17270
Hashimoto	3			167	1044		963					2177
Nodular hyperplasia	1		6		9		206					222
Oxyphilic adenoma	29		24	12	276							341
Thyroiditis-nonspecific	2		91	27	142		174	3				439
Nodular hyperplasia-hyperplastic nodule							3538					3538

Malignant	4		114	12			283	1745	185	11454	931	14728
Anaplastic Ca									185			185
Follicular Ca											931	931
Medullary Ca								1745				1745
Papillary Ca	4		114	12			283			11454		11867

Total	254	388	1248	229	2291	2	22591	1748	185	11457	931	41324

**Table 4:** Cross tabulation of classification results for benign and malignant cell nuclei and colloid structures by the RBF ANN for the training and test sets.

	Benign	Malignant	Total
Training set	13808	6806	20614
Benign	12517	716	13233
Malignant	1291	6090	7381

Test set	13397	7313	20710
Benign	12028	1335	13363
Malignant	1369	5978	7347

Total	27205	14119	41324

**Table 5:** Performance indices for the RBF ANN for the training and test sets and for both sets combined.

	Training set	Test set	Both sets
Sensitivity (%)	82.51	81.37	81.94
Specificity (%)	94.59	90.01	92.29
PPV (%)	89.48	81.74	85.47
NPV (%)	90.65	89.78	90.22
FPR (%)	5.41	9.99	7.71
FNR (%)	17.49	18.63	18.06
OA (%)	90.26	86.94	88.60
PLR	15.25	8.14	10.63
NLR	0.18	0.21	0.20
Odds ratio	82.47	39.34	54.29

**Table 6:** Cross-tabulation of the patient classification subsystem for the numeric and percentages classifiers separately for the training and test sets.

Histology	Numeric classifier		Percentages classifier		Total
	Benign	Malignant	Benign	Malignant
Training set					
Benign	137	8	139	6	145
Malignant	6	73	4	75	79

Test set					
Benign	134	9	136	7	143
Malignant	7	73	4	76	80

Total	284	163	283	164	447

**Table 7:** Performance indices for the patient classification subsystems (numeric and percentages classifiers) for the training and test sets and for both sets combined.

	Numeric classifier			Percentages classifier		
	Training set	Test set	Both sets	Training set	Test set	Both sets
Sensitivity (%)	92.41	91.25	91.82	94.94	95.00	94.97
Specificity (%)	94.48	93.71	94.10	95.86	95.10	95.49
PPV (%)	90.12	89.02	89.57	92.59	91.57	92.07
NPV (%)	95.80	95.04	95.42	97.20	97.14	97.17
FPR (%)	5.52	6.29	5.90	4.14	4.90	4.51
FNR (%)	7.59	8.75	8.18	5.06	5.00	5.03
OA (%)	93.75	92.83	93.29	95.54	95.07	95.30
PLR	16.75	14.50	15.56	22.94	19.41	21.04
NLR	0.08	0.09	0.09	0.05	0.05	0.05
Odds ratio	208.35	155.27	179.03	434.38	369.14	399.28

**Table 8:** Performance indices for the patient classification subsystems (numeric and percentage classifiers) for the training and test sets and for both sets combined.

Reference number	Year of publication	AI method	Number of units	Classification domain	Performance
[81]	1993	A backpropagation ANN and a learning vector quantizer	392 cases	Diagnosis of thyroid function	Overall accuracy on test data subsets was in the range of 96.4–99.7%, when extreme values used for training the overall accuracy were in the range of 92.7–98.8%

[27]	1996	Backpropagation algorithm (three layers)	51 patients	Cell nuclei and patients	Overall accuracy was 90.6% for nuclei classification and 98% on individual patients

[61]	1999	LVQ classifier	198 patients	Benign from malignant thyroid lesions.	Overall accuracy: 97.8%

[31]	2006	Four methods: (1) a linear classifier, (2) a two-layer feedforward ANN, (3) a combined two-layer feedforward ANN generated by the AdaBoost method, and (4) the *k* nearest neighbor classifier (a method with many similarities with LVQ)	157 patients	Benign from malignant thyroid lesions.	(1) 65.17%, (2) 73.20%, (3) 73.20%, and (4) 74.69%

[82]	2007	Backpropagation algorithm (three layers)	197 smears	Follicular carcinomas vs. follicular adenomas	Sensitivity: 97%

[83]	2004	Multilayer perceptron 15 nodes in the input, 1 hidden layer of 15 units, and an output layer	453 patients	High vs. low risk for cancer	Sensitivity: 90.6%, specificity: 62.2%

[84]	2006	Two-layer ANN having inputs as cytological images	30 images from 10 patients for training and 45 patients with follicular adenoma and 39 patients with follicular carcinoma for testing	Follicular carcinomas vs. follicular adenomas	Overall accuracy: 96%

[86]	2008	Multiclassifier system	115 cases	Benign vs. malignant nodules	Overall accuracy: 95.7%

[58]	2011	LVQ ANN	335 cases	In follicular neoplasms suspicious for malignancy and in Hürthle cell tumors	Overall accuracy: 94%

[87]	2014	Optimal transport-based linear embedding for segmented nuclei	94 patients	Distinguishing between follicular lesions	OA LOT-100% except FVPC vs. FC 87%

[89]	2013	Supervised learning-based template matching for segmenting cell nuclei	Microscopy images to segment nuclei	Texture and shape variations of the nuclear structures	Not applicable, used for nuclei segmentation

[90]	2018	ANN model to differentiate FA versus FC on the FNAC material	Microscopy images of FA–FC (26 and 32 cases respectively)	Follicular carcinomas vs. follicular adenomas	Overall accuracy of 93% on image analysis and an overall accuracy of 96% in automatic image classification to differentiate FA and FC

[91]	2018	Convolutional neural network	174 microscopy images	Papillary vs. nonpapillary	Sensitivity: 90.8%, specificity: 83.3%

[92]	2020	Deep learning algorithm for whole slide images (WSIs)	908 whole slide images	Malignancy prediction	Sensitivity: 92%, specificity: 90.5%

## Data Availability

The data used to support the findings of this study are available from the corresponding author upon request.
